# Characterisation of the effect of day length, and associated differences in dietary intake, on the gut microbiota of Soay sheep

**DOI:** 10.1007/s00203-019-01652-w

**Published:** 2019-04-09

**Authors:** Nadine A. Thomas, Andrea M. Olvera-Ramírez, Leticia Abecia, Clare L. Adam, Joan E. Edwards, Georgina F. Cox, Patricia A. Findlay, Elodie Destables, Tracy A. Wood, Neil R. McEwan

**Affiliations:** 1 Rowett Research Institute, Greenburn Road, AB21 9SB Aberdeen, Scotland; 2 Institute of Biological, Environmental and Rural Sciences, Aberystwyth University, Aberystwyth, SY23 3AL Wales; 3 School of Pharmacy and Life Sciences, Robert Gordon University, AB10 7GJ Aberdeen, Scotland; 4 Present Address: Facultad de Ciencias Naturales, Autonomous University of Queretaro, Santiago de Querétaro, Mexico; 5 Present Address: CIC bioGUNE, Bizkaia Science and Technology Park, 48160 Derio, Bizkaia, Spain; 6 Present Address: Laboratory of Microbiology, Wageningen University & Research, 6708 WE Wageningen, The Netherlands

**Keywords:** Soay sheep, Digestive tract, Bacteria, Anaerobic fungi, Ciliated protozoa, Day length

## Abstract

Differences in the rumen bacterial community have been previously reported for Soay sheep housed under different day length conditions. This study extends this previous investigation to other organs of the digestive tract, as well as the analysis of ciliated protozoa and anaerobic fungi. The detectable concentrations of ciliated protozoa and anaerobic fungi decreased with increased day length in both the rumen and large colon, unlike those of bacteria where no effect was observed. Conversely, bacterial community composition was affected by day length in both the rumen and large colon, but the community composition of the detectable ciliated protozoa and anaerobic fungi was not affected. Day length-associated differences in the bacterial community composition extended to all of the organs examined, with the exception of the duodenum and the jejunum. It is proposed that differences in rumen fill and ruminal ‘by-pass’ nutrients together with endocrinological changes cause the observed effects of day length on the different gut microbial communities.

## Introduction

Soays are a breed of sheep that were introduced to Soay Island (part of the St Kilda archipelago off the west coast of Scotland) around 4000 years ago (O’Brien 2000). Due to having adapted to the relatively harsh climate of this island, they are noted as being able to be maintained with minimal husbandry, generally being allowed to exist as semi-feral populations. In turn, this is thought to have contributed to this breed being an example of one of the few which retains many of the traits believed to have been present in the original sheep which were first domesticated.

One of these traits is that the Soays continue to display a high level of photosensitivity; greater than that still seen in most temperate sheep breeds. This retained characteristic is found in many seasonal wild ruminants (e.g. sheep, red deer), which makes Soays of particular interest in nutritional studies, as voluntary food intake increases in summer–spring (long days) and decreases in autumn–winter (short days) even if the quantity of food available is unaffected (Kay 1979). During these longer days, the animals ingest food over a longer period of time, and also eat more rapidly (Rhind et al. 2002), leading to increased rumen capacity and metabolic rate. It is, however, unclear if this corresponds to a change in rumen retention time, as rumen motility is not changed (Rhind et al. 2002).

It is already well documented that a number of factors can influence the microbial composition of the rumen, including diet and feed intake. However, the effects may not alter all populations. For example, a change in dietary intake levels when animals were maintained on longer periods of light (16 h per day) relative to those on shorter days (8 h per day) changed the composition of the rumen bacterial community in Soays, but not that of the ciliated protozoal community (McEwan et al. 2005).

This previous work was restricted to studying the bacteria and ciliated protozoa of the rumen, but did not include analysis of either the anaerobic fungal population or microbes more distally within the tract. Given it is already known that there can be variation in the microbial population in more distal areas of the ruminant digestive tract (e.g. Zeng et al. 2015; Jiao et al. 2016), the work presented here extends the investigation to examine any impact of different daylight periods, and associated differences in dietary intake, to bacteria found in other organs (reticulum, omasum, abomasum, duodenum, jejunum, ileum, caecum and large colon) of the digestive tract, and also to the anaerobic fungal and detectable protozoal population of the rumen and large colon.

## Materials and methods

### Housing of animals and sample collection

Yearling rams (*n* = 12) were removed from a semi-feral flock of Soay sheep at Duthie Farm, Aberdeen, Scotland (57.192°N, 2.208°W) and allocated randomly to one of two treatment groups, either 16 h constant light and 8 h darkness [long day (LD); *n* = 6] or 8 h constant light and 16 h darkness [short day (SD); *n* = 6] conditions. All animals were housed in individual pens for 12 weeks and all procedures received prior approval from the Rowett Research Institute’s Ethical Review Committee. Animals in both groups were given unrestricted access to the same complete diet (comprising g/kg fresh weight): 500 hay, 300 barley, 100 molasses and 90 soybean meal, with vitamin and mineral supplements. Mean dietary intakes during the final 2 weeks of the experiment were recorded. All sheep were approximately 18 months old at the end of the experiment, and were euthanised on a single day using an intravenous overdose of sodium pentobarbitone (Euthesate; Rhone Merieux Ltd, Harlow, Essex, England). Digesta samples were collected immediately postmortem from each animal and stored at − 20 °C until further analysis.

### DNA extraction for bacterial DGGE analysis

Larger plant material was removed from thawed digesta samples by filtering through a double layer of muslin (Eschenlauer et al. 1998). This fraction contained microbes associated with the smaller plant particles, as well as microbes in the liquid fraction. DNA was then isolated from the remaining microbial fraction using a QIAamp^®^ DNA Stool Mini Kit (Qiagen Ltd, West Sussex, England) following the manufacturer’s instructions. Samples were used from nine areas of the digestive tract (rumen, reticulum, omasum, abomasum, duodenum, jejunum, ileum, caecum and large colon) from six animals from each of the 2 days length groups. The total twelve animals were used to permit single-denaturing gradient gel electrophoresis (DGGE) gel analysis of all samples per organ, to avoid potential problems which have been reported previously for inter-gel comparisons with DGGE analyses (e.g. Powell et al. 2005).

### Bacterial DGGE analysis

DGGE analysis was performed as described previously (McEwan et al. 2005). Briefly, analysis was performed as follows. Approximately 200 base pairs (bp) of the V3 region of the 16S *rRNA* gene were amplified using a forward primer: 5ʹ-TAC GGG AGG CAG CAG-3ʹ, and reverse primer: 5ʹ-ATT ACC GCG GCT GCT GG-3ʹ, with a GC-clamp (5ʹ-CGC CCG CCG CGC GCG GCG GGC GGG GCG GGG GCA CGG GGG GCC-3ʹ) at the 5′ terminus of the forward primer. PCR was performed using the following conditions: 5 min at 94 °C; 35 cycles of 1 min at 94 °C, 1 min at 60 °C, 1 min at 72 °C; 1 cycle of 1 min at 94 °C, 1 min at 60 °C, 10 min at 72 °C. Successful amplification was verified and the size of amplicons checked by agarose gel electrophoresis.

A DCode™ Universal Mutation Detection system (16 cm system, BioRad) was used to prepare and run the DGGE gels. DGGE parallel gradient gels ranged from 40 to 60% (8% acrylamide), and were run at 80 mV, 200 mA, for 16 h at 60 °C. DNA was visualised by staining with a DNA Silver Staining Kit (Amersham). In each case, all samples on a gel were from a single-organ source, with no comparisons made between gels.

### DNA extraction for all other PCR-based analysis

Samples of digesta from the rumen and large colon from six animals in each day length group had their dry matter content determined by freeze-drying. The freeze-dried sample material was then ground using a Retsch MM300 Mill. Following grinding, 20 mg of each sample was used for DNA extraction as described previously (Edwards et al. 2008) using a FastDNA Soil Spin Kit (Qbiogene, Carlsbad, California, US) following the manufacturer’s instructions with the exception of an extended bead beating step.

### Real-time PCR of bacterial, ciliated protozoal and anaerobic fungal DNA

Samples were analysed for anaerobic fungal, ciliated protozoal and bacterial DNA content using real-time PCR (RT-PCR), to assess the effect of day length (SD v LD) and gut site (rumen v large colon) on bacterial, protozoal and anaerobic fungal concentrations. For each assay, RT-PCR was performed on an Applied Biosystems 7500 system with each sample analysed in triplicate using 96-well micro-titre plates and a final reaction volume of 25 µl.

Anaerobic fungal RT-PCR was performed using a forward primer: 5ʹ-TTG ACA ATG GAT CTC TTG GTT CTC-3ʹ, and reverse primer: 5ʹ-GTG CAA TAT GCG TTC GAA GAT T-3ʹ that targeted the 5.8S *rRNA* gene (Edwards et al. 2008). Both primers were used at a final concentration of 750 nM. A Taqman probe (5ʹ-FAM-CAA AAT GCG ATA AGT ART GTG AAT TGC AGA ATA CG-TAMRA-3ʹ) was also used at a final concentration of 200 nM. Primers and probe were used with 1X TaqMan Universal PCR master mix (Applied Biosystems). The RT-PCR was performed using the following conditions: enzyme incubation for 2 min at 50 °C; initial denaturation for 10 min at 95 °C; 40 cycles of 15 s denaturation at 95 °C, 1 min primer annealing and extension at 60 °C. Anaerobic fungal DNA standards for the RT-QPCR were prepared as previously described (Edwards et al. 2008).

Bacterial RT-PCR was performed using a SYBR Green assay with a forward primer (5ʹ-GTG STG CAY GGY TGT CGT CA-3ʹ) and reverse primer (5ʹ ACG TCR TCC MCA CCT TCC TC-3ʹ) that targeted the bacterial 16S *rRNA* gene (Maeda et al. 2003). Both primers were used at a final concentration of 400 nM with 1X Power SYBR Green PCR master mix (Applied Biosystems). The RT-PCR was performed under the following conditions: enzyme incubation for 2 min at 50 °C; initial denaturation for 10 min at 95 °C; 40 cycles of 15 s denaturation at 95 °C, 1 min primer annealing and extension at 60 °C. At the end of the assay, a dissociation curve analysis was performed to confirm the specificity of the reaction. Bacterial DNA standards for the RT-QPCR were prepared as previously described (Huws et al. 2010).

Ciliated protozoal RT-PCR was performed using a SYBR Green assay with a forward primer (316f: 5′-GCT TTC GWT GGT AGT GTA TT-3′) and reverse primer (539r: 5′-CTT GCC CTC YAA TCG TWC T-3′) that targeted the ciliated protozoal 18S *rRNA* gene (Sylvester et al. 2004). Both primers were used at a final concentration of 500 nM with 1X Power SYBR Green PCR master mix (Applied Biosystems). The RT-PCR was performed under the following conditions: initial denaturation for 4 min at 94 °C; 45 cycles of 30 s denaturation at 94 °C, 30 s primer annealing at 54 °C and 1 min extension at 72 °C. At the end of the assay, a dissociation curve analysis was performed to confirm the specificity of the reaction. Ciliated protozoal DNA standards for the RT-QPCR were prepared by extracting DNA from a rumen protozoal fraction as previously described (Huws et al. 2009).

### Anaerobic fungal ARISA

Samples were analysed for the composition of anaerobic fungal populations using Automated Ribosomal Intergenic Spacer Analysis (ARISA) (Edwards et al. 2008) to assess the effect of day length (SD v LD) and gut site (rumen v large colon) on anaerobic fungal community composition. The ARISA was performed in triplicate in 96-well micro-titre plates in a final volume of 25 µl, with 1 Unit of *Taq* DNA polymerase (Clontech, Saint-Germain-en-Laye, France) in the manufacturer’s buffer. ARISA PCR products were produced using the forward primer Neo 18S For (5ʹ-AAT CCT TCG GAT TGG CT-3ʹ), and the reverse primer Neo 5.8S REV (5ʹ-CGA GAA CCA AGA GAT CCA-3ʹ). ARISA was performed using the following touchdown PCR conditions: initial denaturation for 5 min at 95 °C; 10 cycles of 95 °C for 30 s (denaturation), 68 °C (− 1 °C touchdown step for each cycle) for 30 s, 72 °C for 30 s (annealing and extension); 25 cycles of 95 °C for 30 s (denaturation), 58 °C for 30 s, and 72 °C for 30 s (annealing and extension) with a final elongation at 72 °C for 6 min and holding of samples at 4 °C. Successful amplification and the size of amplicons were verified by agarose gel electrophoresis. The triplicate PCR products were then pooled, and analysed on an ABI 3100 Genetic Analyser as described previously (Edwards et al. 2008). The data generated were then exported as a table of peaks from Genemapper (Applied Biosystems).

### Data analysis

Mean dietary intakes and final body weights were compared for both LD and SD animals (*n* = 6 for each group) using Student *t* tests, which were supported by prior *F* tests to compare equality of variance.

Hamming distance analysis was performed on samples from individual DGGE gels for bacteria, and ARISA profiles for anaerobic fungi. Hamming distances between individual samples on a DGGE gel were used to determine the number of bands which differed between lanes on the gel. These values were then tabulated in grid format, and the grid was used as the input file for production of a dendrogram using neighbour-joining analysis with PHYLIP software (Felsenstein 1989). *F* test-supported *T* tests were carried out to determine if there was any difference in biodiversity (as assessed by band numbers per lane) between samples collected from sheep housed under either LD or SD conditions. A similar approach was also used for analysis of the ARISA data based on the peaks detected per profile.

RT-QPCR data were analysed on a Log_10_ basis using ANOVA, investigating the effect of day length (SD v LD), organ (rumen v large colon) and day length × organ interaction. This analysis was performed using Genstat statistical software (VSN International Ltd.). Differences were considered significant when *P* values were < 0.05.

## Results

### Feed intakes and final body weight

LD animals had a mean feed intake which was significantly (*P* < 0.001) greater than the SD animals, with 1730 g (SEM = 89.1) of dry matter intake per day compared to 776 g (SEM = 64.3), respectively. There was also a significant (*P* < 0.05) difference in the final live body weight between the two groups: 41.0 kg (SEM = 1.47) for LD versus 36.8 kg (SEM = 1.88) for SD.

### Bacterial community composition

**Fig. 1:**
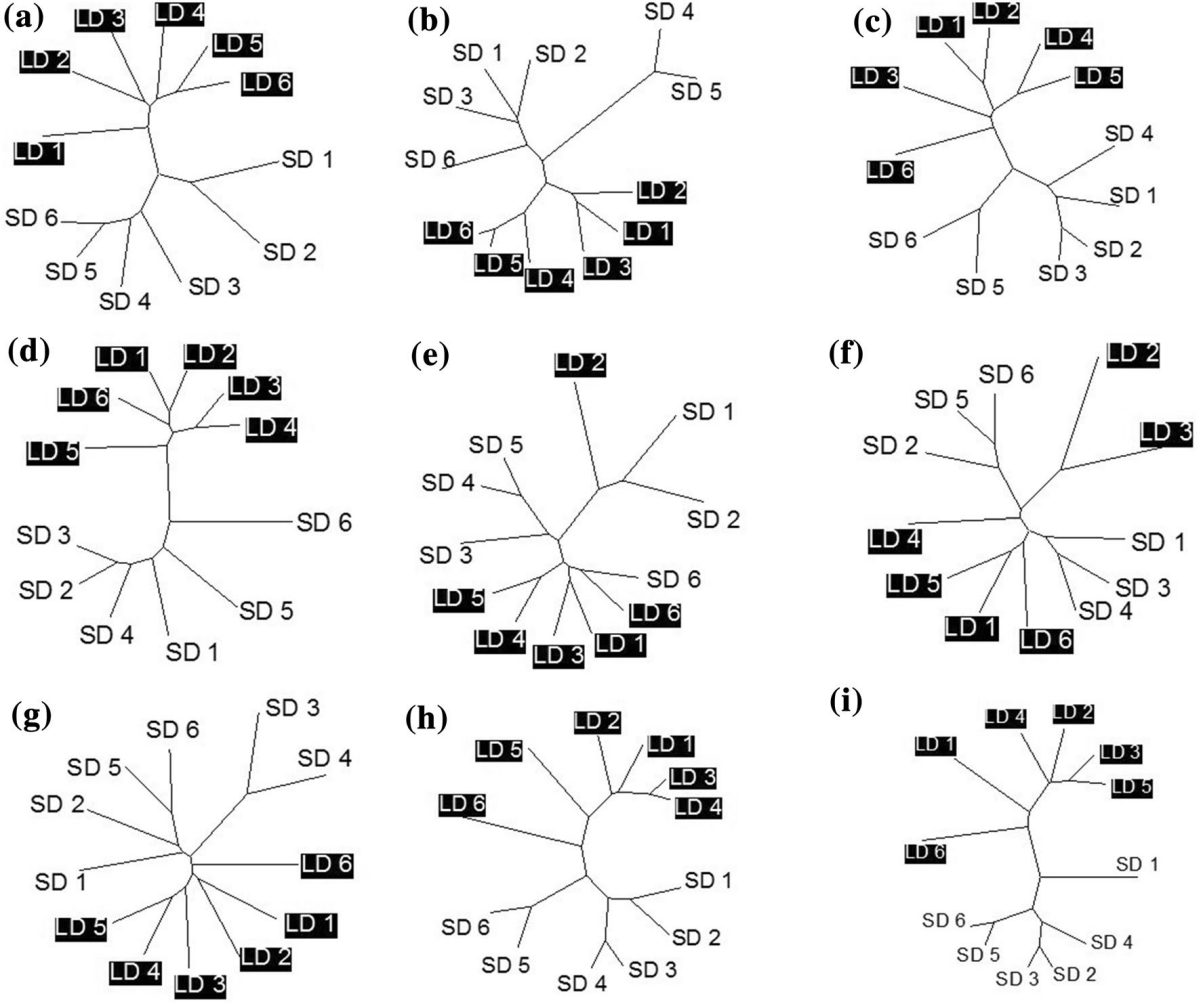
Dendrograms showing the relationship between the bacterial DGGE banding patterns detected in digesta samples obtained from different digestive organs (**a** rumen; **b** reticulum; **c** omasum; **d** abomasum; **e** duodenum; **f** jejunum; **g** ileum; **h** caecum; **i** large colon) in Soay sheep housed under either long (16 h, *n*-= 6) or short (8 h) day lengths (*n* = 6). SD 1 denotes sheep 1 on short days, LD 2 denotes sheep 2 on long days, etc

Hamming distance analyses of the bacterial communities in the different organs isolated from the sheep are shown in Fig. 1. As observed previously (McEwan et al. 2005), day length caused a difference in the rumen bacterial community (Fig. 1a). This difference in bacterial populations was also observed in the other organs of the foregut—the reticulum, omasum and abomasum (Fig. 1b–d). In the duodenum, there is a general trend towards the split being observed (Fig. 1e), although there is a sample from each day length group which clusters in a non-day length-dependent manner. In the jejunum, there is no clear evidence of any day length effect (Fig. 1f). However, in the final three organs of the digestive tract which were studied—ileum, caecum and large colon—the split by day length was again observed (Fig. 1g–i).

*F* test-supported *T* tests to compare biodiversity (i.e. band numbers per lane) showed no significant difference (*P* > 0.05) between samples from LD and SD animals, for all organs with the exception of the abomasum. In the abomasum, there were significantly more (*P* < 0.01) DGGE bands in the samples from LD animals (29.3, SEM = 0.7) relative to the SD animals (21.8, SEM = 2.0).

### Anaerobic fungal community composition

Anaerobic fungal ARISA analysis produced amplicons from both the rumen and large colon samples, with all samples containing peaks within the 365–440 bp range (data not shown). No difference in the composition or biodiversity (i.e. peaks per chromatogram) of samples was detected based on either day length (SD versus LD) or between organs (rumen versus large colon).

### Microbial concentrations

Day length affected the concentrations of anaerobic fungi and ciliated protozoa, with both being decreased in the LD animals compared to the SD (Table 1). Gut site also had a significant effect, with more anaerobic fungi and ciliate protozoa detected in the rumen compared to the large colon (Table 1). These findings were irrespective of whether the data were expressed on a dry weight or wet weight basis (Table 1). In contrast, bacterial concentrations were not affected by either day length or organ (Table 1). No significant day length × gut site was found (Table 1).

**Table 1:** Bacterial, anaerobic fungal and ciliated protozoal DNA concentrations per gram of dry matter (DM) and wet weight (WW) of digesta, together with *P* values for day length (*D*), organ (*O*) and day length × organ (D*O) interaction

	Short day				Long day				*P* values		
	Rumen		Large colon		Rumen		Large colon		*D*	*O*	D*O
Bacteria (mg/g WW)	3.4 ± 0.90		2.8 ± 0.53		2.5 ± 0.95		2.99 ± 0.73		0.473	0.742	0.484
Bacteria (mg/g DM)	18.9 ± 4.89		14.3 ± 1.91		17.5 ± 6.92		18.8 ± 4.41		0.939	0.9895	0.433
Anaerobic fungi (ng/g WW)	328 ± 79.6		1.1 ± 0.26		196 ± 60.1		0.2 ± 0.11		*0.007*	< *0.001*	0.106
Anaerobic fungi (ng/g DM)	1885 ± 455		5.7 ± 1.60		1364 ± 435		1.6 ± 0.7		*0.023*	< *0.001*	0.123
Ciliated Protozoa (mg/g WW)	2.2 ± 0.99		0.002 ± 0.001		0.8 ± 0.22		0.0004 ± 0.0002		*0.012*	< *0.001*	0.286
Ciliated Protozoa (mg/g DM)	12.7 ± 6.08		0.008 ± 0.003		5.3 ± 1.48		0.002 ± 0.001		*0.023*	< *0.001*	0.276

Micro-organism	Short day				Long day				*P* values		
	Rumen		Large colon		Rumen		Large colon				
	Average	SEM	Average	SEM	Average	SEM	Average	SEM	*D*	*O*	D*O
Bacteria (mg/g WW)	3.35	0.90	2.81	0.53	2.51	0.95	2.99	0.73	0.473	0.742	0.484
Bacteria (mg/g DM)	18.87	4.89	14.26	1.91	17.52	6.92	18.82	4.41	0.939	0.985	0.433
Anaerobic fungi (ng/g WW)	328.21	79.58	1.06	0.26	196.05	60.14	0.24	0.11	*0.007*	< *0.001*	0.106
Anaerobic fungi (ng/g DM)	1884.5	454.5	5.7	1.6	1363.7	434.7	1.6	0.7	*0.023*	< *0.001*	0.123
Protozoa (ug/g WW)	2164.8	988.0	1.7	0.7	782.6	218.7	0.4	0.2	*0.012*	< *0.001*	0.286
Protozoa (ug/g DM)	12730.0	6076.2	8.0	2.8	5328.6	1484.9	2.4	1.0	*0.023*	< *0.001*	0.276

## Discussion

Previously it has been shown that there is a difference in the rumen bacterial community composition in sheep relative to the day length conditions under which they were housed (McEwan et al. 2005), but no such difference in the ciliated protozoal populations. Interestingly, the findings presented here also showed no significant differences in the biodiversity of another group of eukaryotic microbes in the rumen; the anaerobic fungi. This observation was true for anaerobic fungal samples from both the rumen and the large colon.

In contrast, day length affected the bacterial community composition in seven of the nine organs examined: rumen, reticulum, omasum, abomasum, ileum, caecum and large colon. The duodenum followed this general trend, but the jejunum had banding patterns which were indistinguishable between the 2-day length groups. However, it is interesting to note that where differences were detected, they were generally not as a consequence of differences in the number of bands, but rather differences in the banding pattern itself. *F* test-supported *T* tests demonstrated that the only organ where there was a significant effect of day length on the number of detectable DGGE bands was the abomasum, where there were more bands in the samples from LD animals than the SD animals (*P* < 0.01).

The abomasum is the most acidic of all of the organs examined in this study (Lee 1977), with the potential to have around 10,000-fold less bacteria than the more densely populated organs such as the rumino-reticulum (Edwards et al. 2005; Simcock et al. 1999). It is in the two organs immediately distal to the abomasum (the duodenum and jejunum) that there is any evidence of failure to differentiate between the two daylight conditions (particularly the jejunum). Again, these are areas of the digestive tract where the microbial concentrations are relatively low (both around 10^6^ bacteria/ml) (Ulyatt et al. 1975).

Despite there being no effect of day length on bacterial concentrations in the rumen or large colon in this study, it has been shown previously that the total and individual concentrations of short-chain fatty acids (SCFAs) were significantly (*P* < 0.001) higher in the rumen of LD animals relative to SD animals (McEwan et al. 2005). This suggests that the ruminal bacteria are more metabolically active in the LD animals, relative to the SD animals. Furthermore, although a lot of the SCFAs are absorbed across the rumino-reticulum wall, it is already known that the SCFAs in the organs lying distal to the rumen may vary depending on dietary factors (Zeitz et al. 2016).

It appears that this effect has less impact on the composition of the jejunum (Fig. 1f), the longest region of the small intestine, which is a major site of nutrient uptake in all mammals. However, in the most distal organ of the small intestine and the organs of the hindgut, which also have a role to play in fermentation of nutrients, the differences between the community structures can be seen once again. It is speculated that this due to the > twofold increase in dietary intake in LD versus SD animals in this study, resulting in more ruminal ‘by-pass’ of nutrients as well as microbial end products reaching distal parts of the gut. Further experiments, however, are needed to confirm this.

Bacterial concentrations in the rumen and large colon were unaffected by day length, unlike the concentrations of the ciliated protozoa and anaerobic fungi. In both the rumen and large colon, increased day length was associated with a decrease in their concentrations. While ciliated protozoa are often regarded as being organisms of the foregut in ruminants, there are a number of examples describing their detection in the hindgut as well (e.g. Mlay et al. 2008). It is suggested that this is due to the longer time needed for these eukaryotic microbes to replicate/reproduce compared to bacteria, resulting in them becoming effectively diluted due to the increased rumen fill over a longer duration of time (Rhind et al. 2002). This effect could in theory also be compounded further down the digestive tract due to the increased volume of digesta leaving the rumen increasing the flow rate through other gut sites. A further effect, associated with decreased rumen retention time in the LD animals compared to the SD animals, is also speculated to occur. The lower concentrations of ciliated protozoa and anaerobic fungi in the large colon, compared to the rumen, are consistent with previous work (Zeitz et al. 2016), although it is unclear how many, if any, of the protozoa were actually living. It is, however, worth noting that *Buxtonella sulcata*, a veterinary important ciliate has been regularly described in the hindgut (e.g. Grim et al. 2015) and that it has described as morphologically similar to *Balantidium coli* (Tomczuk et al. 2005). Therefore, interpretation regarding the existence of ciliate DNA in the hindgut should be interpreted with caution in the context of living protozoa.

The data presented here demonstrate that there is a change in the microbial community in a number of regions of the digestive tract depending on the day length at which the animals were maintained. There was no change in the composition of the diet between the two groups, meaning that the differences were not due to variation in the composition of the diet. In our previous work (McEwan et al. 2005), we attributed the major cause of this to be from the quantity of food ingested. More recently, it has been shown that calorie-restricted diets can impact on the microbiome of individuals, as demonstrated by differences in faecal samples from rats fed on 70% of the ration given to control rats (Fraumene et al. 2018). However, there is also evidence to demonstrate the effects of the gut–brain axis, where there is communication between the brain and the gut microbial community (Cryan and Dinan 2012), and it is already known that there is a hypothalamic response associated with photoperiod in this breed of sheep (Archer et al. 2005). Thus, it is likely that the differences observed in microbial populations are due to some form of combination of calorific intake coupled with endocrinological differences in response to duration of daylight.

In conclusion, this work expands upon the investigation which we previously reported in the rumen of sheep housed under different day length conditions. The concentrations of the ciliated protozoa and anaerobic fungi were affected by day length, unlike the bacteria. Conversely, bacterial community composition was affected by day length, but that of other organisms was not. Day length-associated differences in the bacterial community composition extended to almost all of the organs examined, with the exception of the duodenum and the jejunum. Differences in rumen fill and ruminal ‘by-pass’ nutrients together with endocrinological changes are speculated to cause the observed effects of day length on the gut microbiota.
